# Fluorescent-Antibody Targeting of Insulin-Like Growth Factor-1 Receptor Visualizes Metastatic Human Colon Cancer in Orthotopic Mouse Models

**DOI:** 10.1371/journal.pone.0146504

**Published:** 2016-01-05

**Authors:** Jeong Youp Park, Takashi Murakami, Jin Young Lee, Yong Zhang, Robert M. Hoffman, Michael Bouvet

**Affiliations:** 1 Department of Surgery, University of California San Diego, San Diego, California, United States of America; 2 AntiCancer, Inc., San Diego, California, United States of America; 3 Department of Internal Medicine, Yonsei University College of Medicine, Seoul, Korea; 4 Department of Surgery, Yokohama City University Graduate School of Medicine, Yokohama City, Japan; 5 Surgical Service, VA San Diego Healthcare System, San Diego, California, United States of America; University of Nebraska Medical Center, UNITED STATES

## Abstract

Fluorescent-antibody targeting of metastatic cancer has been demonstrated by our laboratory to enable tumor visualization and effective fluorescence-guided surgery. The goal of the present study was to determine whether insulin-like growth factor-1 receptor (IGF-1R) antibodies, conjugated with bright fluorophores, could enable visualization of metastatic colon cancer in orthotopic nude mouse models. IGF-1R antibody (clone 24–31) was conjugated with 550 nm, 650 nm or PEGylated 650 nm fluorophores. Subcutaneous, orthotopic, and liver metastasis models of colon cancer in nude mice were targeted with the fluorescent IGF-1R antibodies. Western blotting confirmed the expression of IGF-1R in HT-29 and HCT 116 human colon cancer cell lines, both expressing green fluorescent protein (GFP). Labeling with fluorophore-conjugated IGF-1R antibody demonstrated fluorescent foci on the membrane of colon cancer cells. Subcutaneously- and orthotopically-transplanted HT-29-GFP and HCT 116-GFP tumors brightly fluoresced at the longer wavelengths after intravenous administration of fluorescent IGF-1R antibodies. Orthotopically-transplanted HCT 116-GFP tumors were brightly labeled by fluorescent IGF-1R antibodies such that they could be imaged non-invasively at the longer wavelengths. In an experimental liver metastasis model, IGF-1R antibodies conjugated with PEGylated 650 nm fluorophores selectively highlighted the liver metastases, which could then be non-invasively imaged. The IGF-1R fluorescent-antibody labeled liver metastases were very bright compared to the normal liver and the fluorescent-antibody label co-located with green fluorescent protein (GFP) expression of the colon cancer cells. The present study thus demonstrates that fluorophore-conjugated IGF-1R antibodies selectively visualize metastatic colon cancer and have clinical potential for improved diagnosis and fluorescence-guided surgery.

## Introduction

Colorectal cancer is the second leading cause of cancer-related deaths in Western countries [1]. Development of colonoscopy enables early detection and removal of precancerous adenomatous polyps [2,3]. Complete surgical resection can cure well selected patients with liver metastasis [4,5]. Improved imaging of metastatic colon cancer should increase survival by making colonoscopy and surgery more effective. We have previously shown that targeting orthotopic metastatic colon cancer, including patient-derived orthotopic xenografts (PDOX) models, with fluorescent anti-carcinoembyronic antigen (CEA) antibodies enabled improved tumor visualization and effective fluorescence-guided surgery (FGS) [6]. Targeting of the epidermal growth factor receptor with fluorescent antibodies improved colonoscopy [7].

Type I insulin-like growth factor receptor (IGF-1R) is a transmembrane tyrosine kinase receptor comprising two α and two β chains and is the major receptor for IGF-I and IGF-II. IGF-1R is expressed in 51~100% of the colon cancers depending on the study [8–10]. The high frequency of expression in colon cancer and membrane subcellular location make IGF-1R a potential target for fluorescent antibodies to enable cancer visualization, diagnosis, and FGS [11]. Using IGF-1R also has additional clinical potential since the overexpression of IGF-1R correlates with shorter median survival of colon cancer patients who undergo surgery and adjuvant chemotherapy [10].

The present report demonstrates the feasibility of IGF-1R targeted fluorophore-conjugated antibodies to visualize metastatic colon cancer in appropriate mouse models.

## Materials and Methods

### Colon cancer cell lines

The human colon cancer cell lines HT-29 [12] and HCT 116 [13] were maintained in RPMI 1640 medium supplemented with 10% fetal bovine serum (Hyclone, Logan, UT), penicillin/streptomycin (Gibco-BRL, Carlsbad, CA), sodium pyruvate (Gibco-BRL), sodium bicarbonate (Cellgro, Manassas, VA), L-glutamine (Gibco-BRL), and minimal essential medium nonessential amino acids (Gibco-BRL). All cells were cultured at 37°C in a 5% CO_2_ incubator.

### Construction of GFP-expressing colon cancer cell line

The construction of green fluorescent protein (GFP) expressing HCT 116 is previously described [14–16]. For GFP gene transduction, 20% confluent HCT 116 cells [17] were incubated with a 1:1 mixture of retroviral supernatants of the PT67 packaging cells and RPMI 1640 (Gibco-BRL, Life Technologies, Inc.) for 72 h. The cells were harvested by trypsin/EDTA 72 h after incubation with GFP retroviral supernatants and subcultured at a ratio of 1:15 into selective medium that contained 200 μg/ml G418. The level of G418 was increased to 800 μg/ml stepwise. Clones expressing GFP were isolated with cloning cylinders (Bel-Art Products, Pequannock, NJ) by trypsin/EDTA and were amplified and transferred by conventional culture methods. High GFP expression clones were then isolated in the absence of G418 for > 10 passages to select for stable expression of GFP [14–16].

### Mice

Athymic nu/nu nude mice (AntiCancer Inc., San Diego, CA), 4–6 weeks old, were used in this study. Mice were kept in a barrier facility under HEPA filtration. Mice were fed with an autoclaved laboratory rodent diet. All mouse surgical procedures and imaging were performed with the animals anesthetized by intramuscular injection of 50% ketamine, 38% xylazine, and 12% acepromazine maleate (0.02 ml). Animals received buprenorphine (0.10 mg/kg ip) immediately prior to surgery and once a day over the next 3 days to ameliorate pain. The condition of the animals was monitored every day. Tumors were measured with calipers every other day. When tumors became larger than 2 cm or a deep ulcer was formed, euthanasia was performed. The animals were all sacrificed 2–3 weeks after surgery. CO_2_ inhalation was used for euthanasia. To ensure death following CO_2_ asphyxiation, cervical dislocation was performed. All animal studies were approved by AntiCancer, Inc.’s Institutional Animal Care and Use Committee (IACUC) in accordance with the principals and procedures outlined in the National Institute of Health Guide for the Care and Use of Animals under Assurance Number A3873-1.

### Antibody-dye conjugation

Mouse monoclonal antibodies to IGF-1R (clone 24–31; Thermo Scientific, Rockford, IL, USA) were conjugated with DyLight 650, Dylight 550, or PEGylated Dylight 650 dyes (Thermo Scientific, Rockford, IL, USA) per manufacturer specifications, ensuring a minimum of at least 4:1 dye: protein ratio [18]. Protein:dye concentrations were confirmed using a NanoDrop Spectrophotometer (Thermo Fisher Scientific, Waltham, Massachusetts) [18].

### Western blotting

Cell lysates were extracted in lysis buffer containing 70 mM β-glycerophosphate, 0.6 mM sodium orthovanadate, 2 mM MgCl_2_, 1 mM ethylene glycol tetraacetic acid, 1 mM DTT (Invitrogen, Grand Island, NY, USA), 0.5% Triton-X100, 0.2 mM phenylmethylsulfonyl fluoride, and 1% protease inhibitor cocktail (Sigma-Aldrich, St. Louis, MO, USA). Lysates were separated by sodium dodecylsulfate–polyacrylamide gel electrophoresis (SDS-PAGE) and transferred to polyvinylidene fluoride membranes (Millipore, Billerica, MA, USA). The membranes were blocked in 5% (w/v) non-fat dry milk and probed with anti-IGF-1Rα (SC-712, Santa Cruz, Dallas, TX, USA). The immunoreactive proteins were visualized using the SuperSignal West Pico Chemiluminescent Substrate (Thermo Scientific, Rockford, IL, USA).

### Labeling of live cells in vitro using fluorescent IGF-1R antibodies

HT-29 and HCT 116 cells (2 x 10^5^) were cultured overnight. IGF-1R antibody (clone 24–31) conjugated with DyLight 550 dye was diluted to 4 μg/ml in phosphate-buffered saline (PBS, Corning cellgro, Manassas, VA). The culture medium was aspirated from the cells and the diluted antibody was added to the live cells. Cells were incubated with antibody for 1 hour at room temperature. The cells were washed gently 2 times with PBS after the antibody was aspirated. The cells were observed under an FV1000 confocal microscope (Olympus, Tokyo, Japan) with white light and 559 nm laser [19].

### Immunohistochemistry

Anti-IGF-1R (clone 24–31) conjugated with DyLight 650 was used for staining tumor sections on slides. The slides were incubated with 10% normal donkey serum for 1 hour at room temperature, and incubated with the conjugated antibody at room temperature for 1 hour at a dilution of 1:100. Tissues were dried and observed with an IV-100 scanning laser microscope (Olympus, Tokyo, Japan) with a 633 nm laser [20]. Alternate slides from the same frozen tumor tissue were stained with hematoxylin and eosin and observed under light microscopy.

### Subcutaneous and orthotopic tumor mouse models

HT-29-GFP and HCT 116-GFP human colon cancer cells (2 × 10^6^) were injected subcutaneously into the flanks of nude mice. When the subcutaneous tumors grew between 10 and 20 mm in diameter, they were harvested and fragmented into small fragments. The tumor fragments were implanted orthotopically in colon of nude mice, as previously described [6,21,22]. Briefly, a single 3-mm^3^ tumor fragment, which was suitable for suturing and also resulted in reproducible tumor growth, was attached to the mesenteric border of the cecal wall using 8–0 nylon surgical sutures (Ethilon™; Ethicon Inc., Somerville, NJ). On completion, the cecum was returned to the abdomen, and the incision was closed in one layer using 6–0 nylon surgical sutures (Ethilon).

### Liver metastasis model

HCT116-GFP colon cancer cells (2 x 10^6^), with matrigel, were injected into the spleen of nude mice as previously described [17]. Liver metastases appeared over a period of 2–3 weeks.

### Imaging

In both subcutaneous and orthotopic tumor models, the mice were injected with the IGF-1R antibody, conjugated with fluorophore into the tail vein, and then whole body non-invasive imaging was performed using the OV100 Small Animal Imaging System (Olympus, Tokyo, Japan) as described above [23]. The optimal dose of fluorophore-conjugated IGF-1R antibody was determined by the dose that produced images with the best tumor-to-background ratio in the subcutaneous tumor model as directly visualized with the OV100. After whole body non-invasive imaging in the orthotopic tumor model, ex vivo imaging of the tumor with the OV-100 was performed at the end of the experiment. In the liver orthotopic-transplant model, after ensuring the presence of liver metastases by non-invasive whole body imaging with the OV100, the mice were injected with anti-IGF-1R (clone 24–31) conjugated to PEGylated DyLight 650. The mice were sacrificed 24 hours after injection and a ventral vertical incision from the sternum to the lower abdomen was made and then the liver was imaged using the OV-100. The entire liver with metastasis was taken out and ex vivo imaging with the OV-100 was repeated. The background signal in native liver was reduced by the choice of excitation filters. GFP was excited by a 460–490 nm filter and the fluorescent antibody was excited with a 595–635 nm filter.

### Image analysis

Image processing was done with Adobe Photoshop CS3 (Adobe Systems Inc., San Jose, California).

## Results

### Expression of IGF-1R in colon cancer cells in vitro

**Fig 1:**
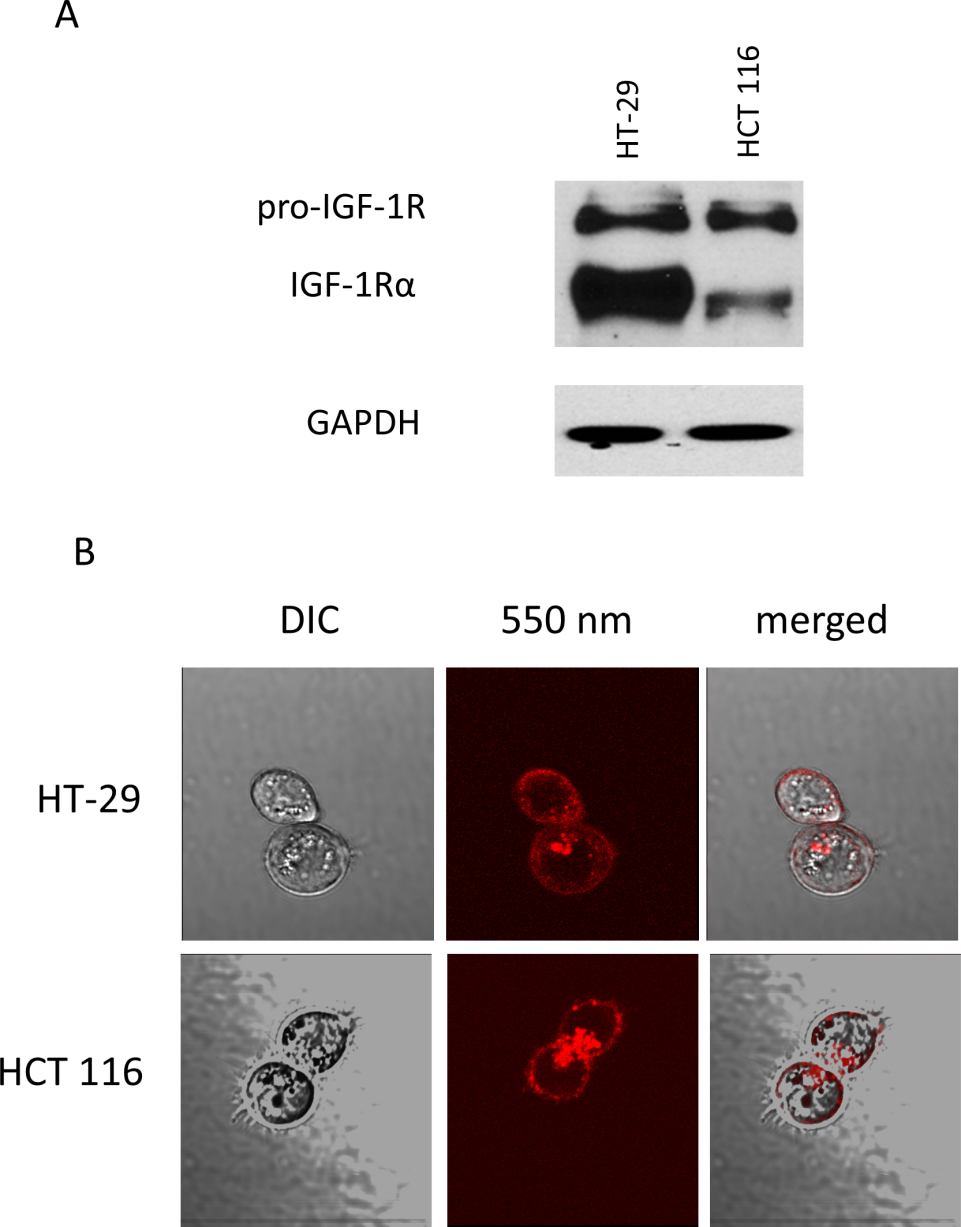
Characterization of colon cancer cell lines. (A) Western blot analysis shows pro-IGF-1R and IGF-1Rα expression at 200 and 130 kDa in colon cancer cell lines, respectively (HT-29 and HCT 116). (B) Labeling of live HCT 116 and HT-29 cells with 550 nm fluorophore-conjugated antibodies shows multiple fluorescent foci on the surface. Representative fluorescence images merged with corresponding DIC (differential interference contrast) images (x60 water immersion objective with the FV1000, using the 559 nm laser).

Both HT-29-GFP and HCT 116-GFP colon cancer cell lines expressed pro-IGF-1R and IGF-1R, as determined with Western blotting. HT-29-GFP expressed IGF-1R more strongly than HCT-116-GFP (Fig 1A). The Western blotting was repeated three times, and we observed the same results each time. After incubation with the fluorescent conjugated IGF-1R antibody, without permeation, multiple fluorescent foci were visualized on the HT-29 and HCT 116 cells in monolayer culture, as observed under fluorescence microscopy (Fig 1B). Differential interference contrast microscopy confirmed that the conjugated antibody reacted with IGF-1R on the surface of the cancer cells (Fig 1B).

### Targeting subcutaneous colon tumors in nude mice with fluorescent IGF-1R antibodies

**Fig 2:**
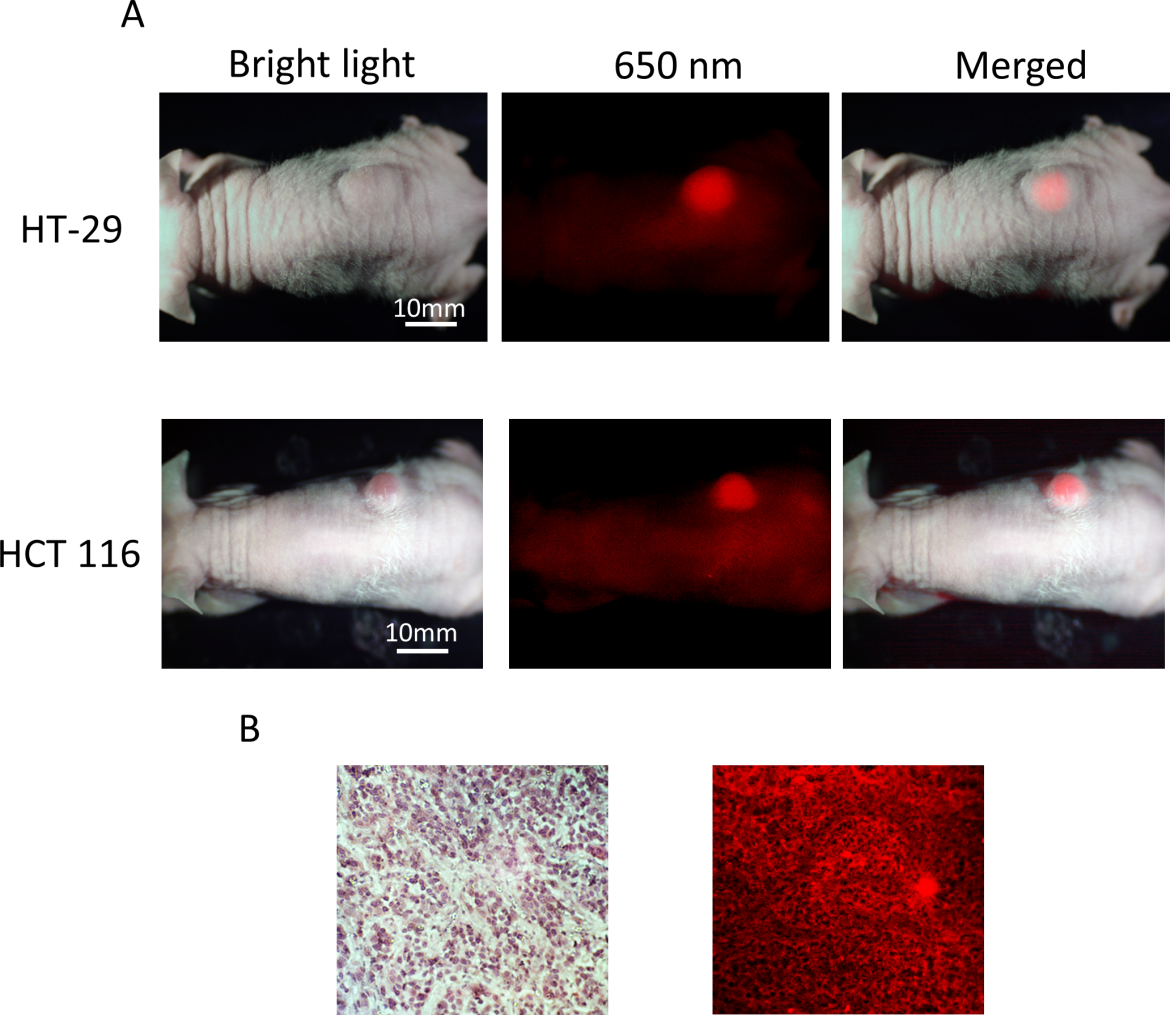
Imaging of 650 nm fluorophore-conjugated IGF-1R antibody targeting of subcutaneous colon tumors in vivo. (A) The mouse that is imaged under both white light and fluorescence illumination. The intensity of fluorescence from the HCT 116 and HT-29 subcutaneous tumors is much greater than background. (B) Hematoxylin & eosin staining (x200, left). Fluorescence immunostaining for IGF-1R (x20 regular objective, IV-100 scanning laser microscope (Olympus) with a 633 nm laser, right) of frozen HCT 116 tumor samples shows fluorescence on the membrane of the cancer cells.

When HT-29-GFP or HCT 116-GFP subcutaneous tumors reached approximately 10 mm in diameter, the animals were each given a single 10, 30, and 50 μg dose of DyLight 650-conjugated anti-IGF-1R (clone 24–31) in 150 μl PBS via the tail vein. Subcutaneous tumor imaging was done when the tumor diameter was approximately 10 mm. The mice were imaged by both bright-field and fluorescence illumination using the OV100 after 48 hours. With 30 μg dose of conjugated anti-IGF-1R, which produced the best contrasted images, both HT-29 and HCT 116 subcutaneous tumors had stronger fluorescence compared to background (Fig 2A). Fluorescence immunostaining was performed on frozen tumor samples from HCT 116 tumors and confirmed the presence of IGF-1R on the cancer cell membranes (Fig 2B).

### Targeting orthotopic colon tumors in nude mice with fluorescent IGF-1R antibodies

**Fig 3:**
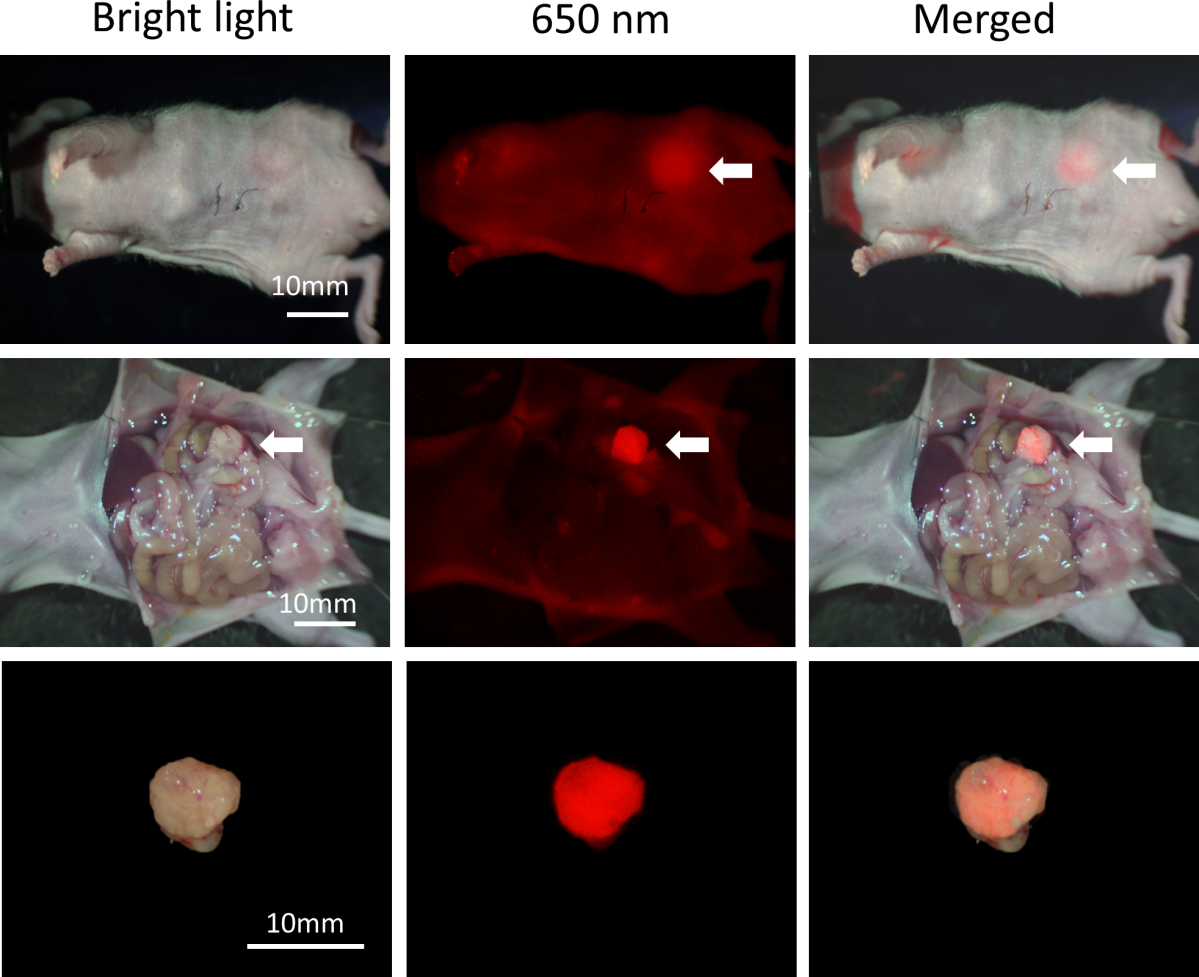
Imaging of IGF-1R targeting of orthotopically-transplanted HCT 116 colon tumors in vivo. Fluorescence from orthotopically transplanted colon tumors at the cecum was detected before and after abdominal laparotomy. Also, fluorescence from the resected tumor was detected. Weak fluorescence was also detected from the skin and, bladder and intestinal contents, but at much lower intensity than the tumor. White arrows indicate colon tumor.

Mice with tumors orthotopically-implanted in the mouse cecum were injected with DyLight 650-conjugated anti-IGF-1R (clone 24–31) antibody with a single 30 μg dose via the tail vein 14 days after tumor implantation. Imaging was performed before and after opening the abdomen of the mice. Tumor size was approximately 10 mm. The OV100 detected tumor fluorescence non-invasively, by intravital open imaging as well as ex-vivo (Fig 3). Fluorescence was observed in the skin, bladder, and intestinal contents, but at low intensity.

### Targeting of colon-cancer liver metastasis in nude mice with fluorescent IGF-1R antibody

**Fig 4:**
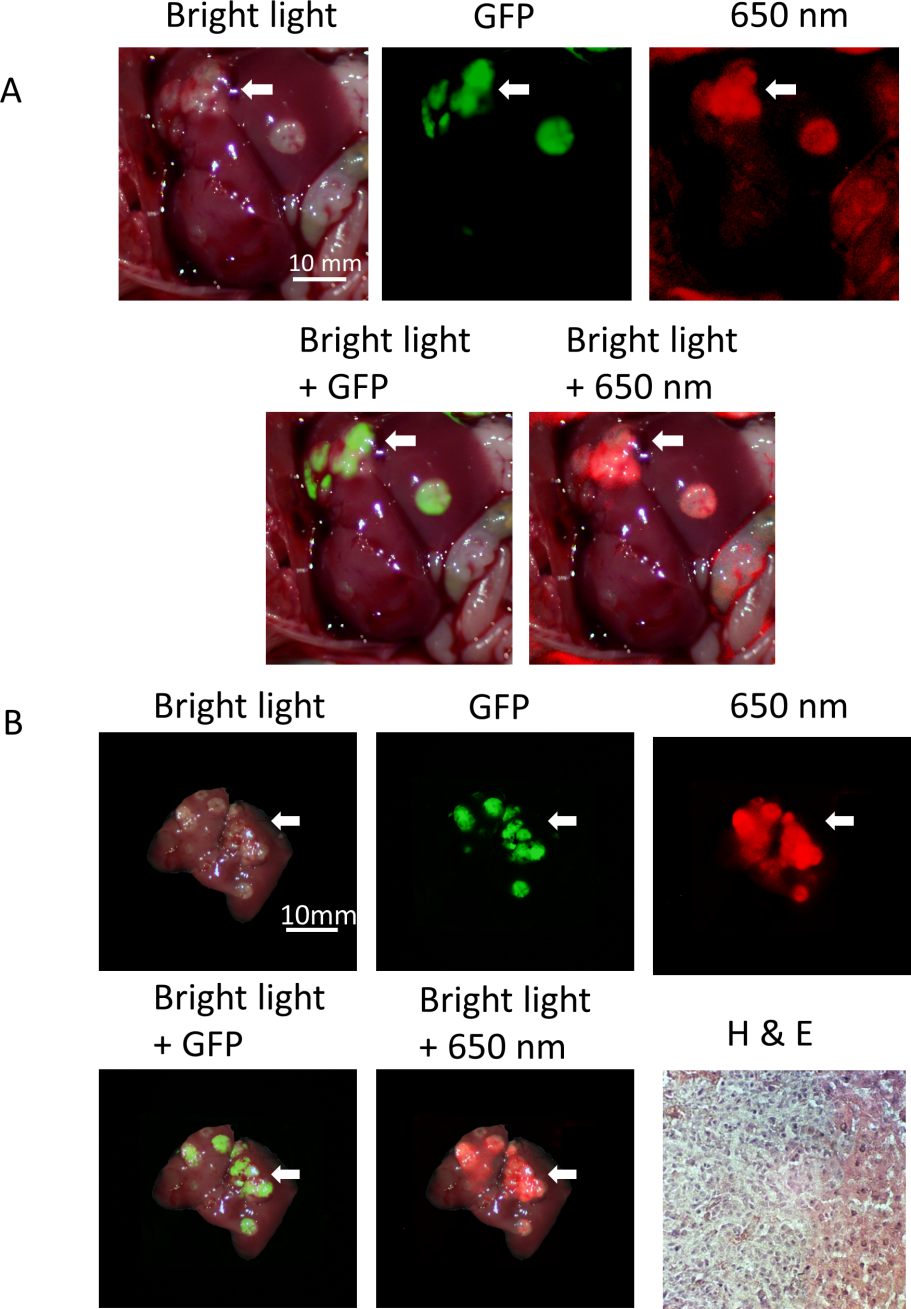
Imaging of IGF-1R targeting of liver metastasis. Anti-IGF-1R conjugated to PEGylated 650 nm dye selectively labeled the HCT 116-GFP metastatic tumors. Both in vivo (A) and ex vivo (B) imaging show that the 650 nm fluorophore-conjugated IGF-1R antibodies co-localized with HCT 116-GFP fluorescence and more accurately demarked the tumor compared to bright light imaging (white arrowheads). H & E staining of tissue sample (x200) expressing fluorescence confirms the presence of metastatic tumor in the mouse liver.

Three weeks after injecting HCT 116-GFP cells injected into the mouse spleen, the presence of liver metastases was observed by non-invasive whole body imaging. The mice were then treated with PEGylated DyLight 650-conjugated anti-IGF-1R (clone 24–31) with a single 90 μg dose via the tail vein and sacrificed 24 hours after antibody injection. We did imaging within 24 hours in the liver metastasis model because our previous experience showed that conjugated antibody detached from the tumor more rapidly in the liver metastasis model compared to the subcutaneous and orthotopic tumor models [24]. Ventral abdominal laparotomy of the mice demonstrated multiple metastatic tumors in the liver. GFP and 650 nm fluorescence originated from metastatic tumors, with normal liver only showing a very weak signal (Fig 4A). Although it was not possible to count all the very small tumors, ex vivo imaging also showed that the 650 nm signal co-localized with GFP and demarcated the extent of the tumor more accurately compared to bright light imaging. Hematoxylin & eosin staining of tissue samples expressing fluorescence confirmed the presence of metastatic tumors in the mouse liver (Fig 4B).

## Discussion

Targeting IGF-1R in sarcoma with radiolabeled antibody and breast cancer with fluorophore-conjugated antibody has been reported [11,25]. In the present study, antibody targeting the alpha subunit of IGF-1R was used. In vitro imaging confirmed that the conjugated antibody bound to IGF-1R on the outer membrane of colon cancer cells. Fluorescently-conjugated anti-IGF-1R antibodies made the tumor more fluorescent than background, easily distinguishing tumor from surrounding normal organs. The present report suggests that fluorescence imaging with fluorophore-conjugated IGF-1R antibody could be combined with endoscopy and FGS for better diagnosis and treatment of colon cancer.

IGF-1R targeted imaging may be able to detect high risk polyps as suggested by our results and by previous reports that IGF-1R expression was detected in colon adenomas and became stronger as the degree of carcinogenesis progressed. Non-neoplastic hyperplastic polyps did not express IGF-1R [26]. Predicting the carcinogenic risk of premalignant polyps with fluorescent IGF-1R antibodies can enable individualized treatment for colon polyps, even without tissue sampling and can help avoid unnecessary invasive procedures or repeated treatment.

IGF-1R targeted therapy is being developed and tested in clinical trials [27]. Determining the expression of IGF-1R in tumors with fluorescence imaging prior or during treatment might be able to guide treatment or predict treatment response. Fluorescence imaging has advantages since it does not require invasive tumor sampling and can be repeated easily [28].

We have previously demonstrated effective FGS of colon cancer in mouse models using fluorescent anti-CEA antibodies [6,29,30]. Fluorescence imaging has been shown to detect tiny liver metastases and remove them later by surgery in patients [31]. Especially with colon cancer, detecting liver metastasis with fluorescence imaging is important since it can enhance complete resection. Our results showed that with IGF-1R antibody conjugated with a PEGylated fluorophore, the metastases became much more fluorescent compared to the normal liver background, and became more accurately detected [24]. We previously showed that PEGylated dyes conjugated to tumor-specific antibodies reduced non-specific uptake by normal liver [24]. The goal of the present manuscript was to demonstrate that a fluorescent antibody to IGF-1R could identify the extent of liver metastasis in orthotopic models of colon cancer better than standard bright light visualization, and we achieved this goal. Fluorescence imaging using fluorophore-conjugated IGF-1R antibody of liver metastasis should be effective for FGS.

In conclusion, we have shown that fluorophore-conjugated IGF-1R antibody is very effective in labeling colon cancer in mouse models. Formal bio-distribution analysis and further studies with colon cancer PDOX models are needed to confirm that IGF-1R targeted imaging can be used in clinical settings. Our previous studies using fluorophore conjugated antibodies to CEA, CA 19–9, Kit, and MUC-1 have demonstrated their capability of brightly labeling gastrointestinal tumors in vivo [6,24,30,32–40]. Imageable biomarkers will have important clinical potential for diagnosis and therapies such as FGS.
